# Evaluation of a novel CBCT conversion method implemented in a treatment planning system

**DOI:** 10.1186/s13014-023-02378-2

**Published:** 2023-11-16

**Authors:** Wolfgang Lechner, Dávid Kanalas, Sarah Haupt, Lukas Zimmermann, Dietmar Georg

**Affiliations:** Division of Medical Radiation Physics, Department of Radiation Oncology, Medical University of Vienna/AKH Vienna, Währinger Gürtel 18-20, 1090 Vienna, Austria

**Keywords:** CBCT based dose calculation, Deformable registration, Adaptive radiotherapy, Accuracy

## Abstract

**Background:**

To evaluate a novel CBCT conversion algorithm for dose calculation implemented in a research version of a treatment planning system (TPS).

**Methods:**

The algorithm was implemented in a research version of RayStation (v. 11B-DTK, RaySearch, Stockholm, Sweden). CBCTs acquired for each ten head and neck (HN), gynecology (GYN) and lung cancer (LNG) patients were collected and converted using the new algorithm (CBCT_c_). A bulk density overriding technique implemented in the same version of the TPS was used for comparison (CBCT_b_). A deformed CT (dCT) was created by using deformable image registration of the planning CT (pCT) to the CBCT to reduce anatomical changes. All treatment plans were recalculated on the pCT, dCT, CBCT_c_ and the CBCT_b_. The resulting dose distributions were analyzed using the MICE toolkit (NONPIMedical AB Sweden, Umeå) with local gamma analysis, with 1% dose difference and 1 mm distance to agreement criteria. A Wilcoxon paired rank sum test was applied to test the differences in gamma pass rates (GPRs). A *p* value smaller than 0.05 considered statistically significant.

**Results:**

The GPRs for the CBCT_b_ method were systematically lower compared to the CBCT_c_ method. Using the 10% dose threshold and the dCT as reference the median GPRs were for the CBCT_c_ method were 100% and 99.8% for the HN and GYN cases, respectively. Compared to that the GPRs of the CBCT_b_ method were lower with values of 99.8% and 98.0%, for the HN and GYN cases, respectively. The GPRs of the LNG cases were 99.9% and 97.5% for the CBCT_c_ and CBCT_b_ method, respectively. These differences were statistically significant. The main differences between the dose calculated on the CBCTs and the pCTs were found in regions near air/tissue interfaces, which are also subject to anatomical variations.

**Conclusion:**

The dose distribution calculated using the new CBCT_c_ method showed excellent agreement with the dose calculated using dCT and pCT and was superior to the CBCT_b_ method. The main reasons for deviations of the calculated dose distribution were caused by anatomical variations between the pCT and the corrected CBCT.

**Supplementary Information:**

The online version contains supplementary material available at 10.1186/s13014-023-02378-2.

## Introduction

The primary aim of image guided radiotherapy (IGRT) is to provide (volumetric) information of the patient’s anatomy which is currently mainly used for correcting and validating the treatment position of the patient. This information can also be used in adaptive radiotherapy (ART) concepts to account for anatomical changes occurring at different points in time during the course of a treatment [1–4]. In recent years, MR imaging for IGRT has gained importance due to the higher soft tissue contrast compared to CT images and allows dedicated online-ART workflows [5–7]. Recently treatment units which support streamlined online-ART workflows based on X-ray images became commercially available [8–10]. However, standard medical linear accelerators producing photon beams are still the most widely used machine type in modern radiation oncology. Enabling online-ART concepts for this machine type requires fast and accurate dose calculation on cone beam CT (CBCT) images.

The image quality of CBCT is often lower compared to fan-beam CT and the Hounsfield Units (HU) to mass- or electron density conversion cannot be directly converted between these two modalities due to the different amount of scattered radiation and the projection geometry [11, 12]. Several groups have investigated different strategies to overcome this limitation of the CBCT images and showed that accurate dose calculation is feasible [13–23].

In this work, we have investigated an algorithm providing dose calculation with high accuracy implemented in a research version of a commercial treatment planning system (TPS). Two scenarios were tested on three different body sites using the dose calculated on the planning CT as ground truth. First, the planning CT images were deformably registered to the CBCT to minimize anatomical differences to focus on the accuracy of the dose calculation. Second, the dose calculated on CBCT was compared to the dose calculated on the original CT to show which variation can be expected in a realistic scenario. The results of the new algorithm were benchmarked against the standard algorithm implemented in the TPS.

## Materials and methods

### Patient cohort and image acquisition

For this study, ten head and neck (HN), ten gynecological (GYN) and ten lung (LNG) cancer patients treated with IGRT using CBCT were retrospectively selected. The HN and GYN patients received volumetric modulated arc treatment (VMAT) using either 6 MV or 10 MV photon beams with doses up to 70 Gy in 35 fractions (HN) and 45 Gy in 25 fractions (5 fractions/week) prescribed at the median dose of the high risk PTV (GYN), respectively. LNG patients received stereotactic body radiation therapy using 3D-conformal radiotherapy using 10 MV FFF beams with doses of 60 Gy in eight fractions or 45 Gy in three fractions prescribed to the 65% isodose covering 99% of the PTV. The planning CTs (pCT) of the patients were acquired with a Somatom AS (Siemens AG, Forchheim, Germany) CT using a slice thickness of 2–4 mm depending on the treatment. All images were reconstructed using iMAR (Iterative metal artifact reduction) if necessary due to implants or dental fillings. The patients were treated using VersaHD linear accelerators (Elekta AB, Stockholm, Sweden). Consequently, CBCT image data was acquired using the XVI kV imaging system (Elekta, Stockholm, Sweden). The standard protocols provided by Elekta were used in all cases, i.e. ‘Fast Head and Neck S20’, ‘Pelvis M20’, ‘Chest M20’ presets were used for the acquisition of the CBCT data for the HN, GYN and LNG patients, respectively. The CBCTs were using the same slice thickness as the pCT.

### Algorithms

All deformations and dose calculations were done in a research version of the RayStation TPS (V. 11B-DTK, RaySearch Laboratories, Stockholm, Sweden) and dose calculation was performed using its collapsed cone algorithm.

The Analytical Constraining Deformation Algorithm (ANACONDA) implemented in the TPS was used to facilitate all deformable image registrations applied in this study. This algorithm calculates the deformation vector field based on the image intensities. Two types of regions of interest (ROIs) can be used to guide the algorithm, i.e. controlling ROIs can be contoured on both image sets to optimize the deformation, while focus ROIs can be used to restrict the deformation to a specified region. Due to the limited image quality of the CBCT, the use of controlling ROIs was omitted and only focus ROIs were used. The FOV of the CBCT was contracted by 2 cm and the resulting structure was used as focus ROI to avoid unreasonable deformations near the edge of the FOV.

The novel CBCT correction algorithm is similar to an algorithm proposed by *Shi *et al*.* which also uses deformable image registration of the pCT to the CBCT for the correction of image intensities and shading artefacts of the CBCT [24]. In short, the algorithm calculates a correction map based on the differences between the CBCT and the pCT. This correction map is used to enhance the quality of the CBCT. Further, the algorithm analyses the image intensities of different tissue types in the CBCT and the pCT and calculates a calibration curve using piecewise linear interpolation between these tissue types. Additionally, the algorithm employs a stitching technique to simulate missing tissue outside the field of view (FOV) of the CBCT by attaching the pCT outside the FOV [20, 21]. Early versions of the algorithm using the scripting interface of the TPS have been tested have been tested by other groups [21–23]. The output of the algorithm is a so called corrected CBCT (CBCT_c_) which can be used for dose calculation using the CT to mass-density conversion curve of the pCT.

In addition to the novel algorithm, the standard algorithm for CBCT dose calculation implemented in the TPS was used for comparison. This algorithm uses a threshold-based bulk density overriding technique. Six different tissues-types can be segmented based on the image intensity. A standard density is assigned to these segmented tissue types. The thresholds of the individual tissue types need to be adjusted for each image to yield optimal calculation results. The output of this algorithm is a step-wise function converting CBCT image intensities to mass density to enable dose calculation. Dose distributions calculated using this method are labeled CBCT_b_.

### Analysis

**Fig. 1:**
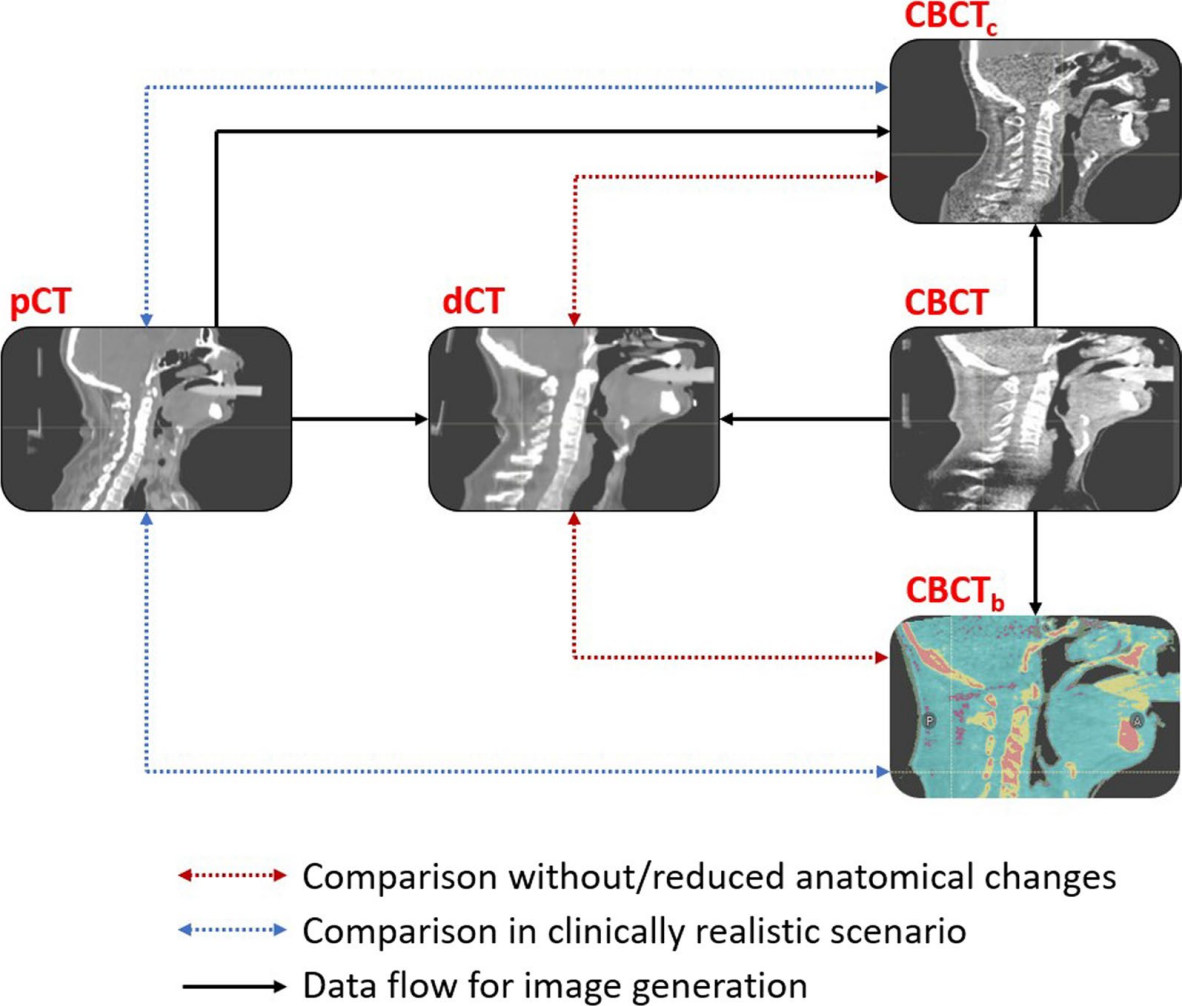
Workflow of the data analysis. The planning CT (pCT) was deformably registered to the CBCT to generate the dCT which has reduced anatomical differences with respect to the CBCT compared to the pCT. The CBCT_c_ was generated using the acquired CBCT. For the CTBCT_b_ a step-wise HU to mass-density conversion curve was calculated. The dose calculated on the CBCT_c_ and CTBCT_b_ were compared to the dose calculated on the pCT and the dCT, respectively

**Fig. 2:**
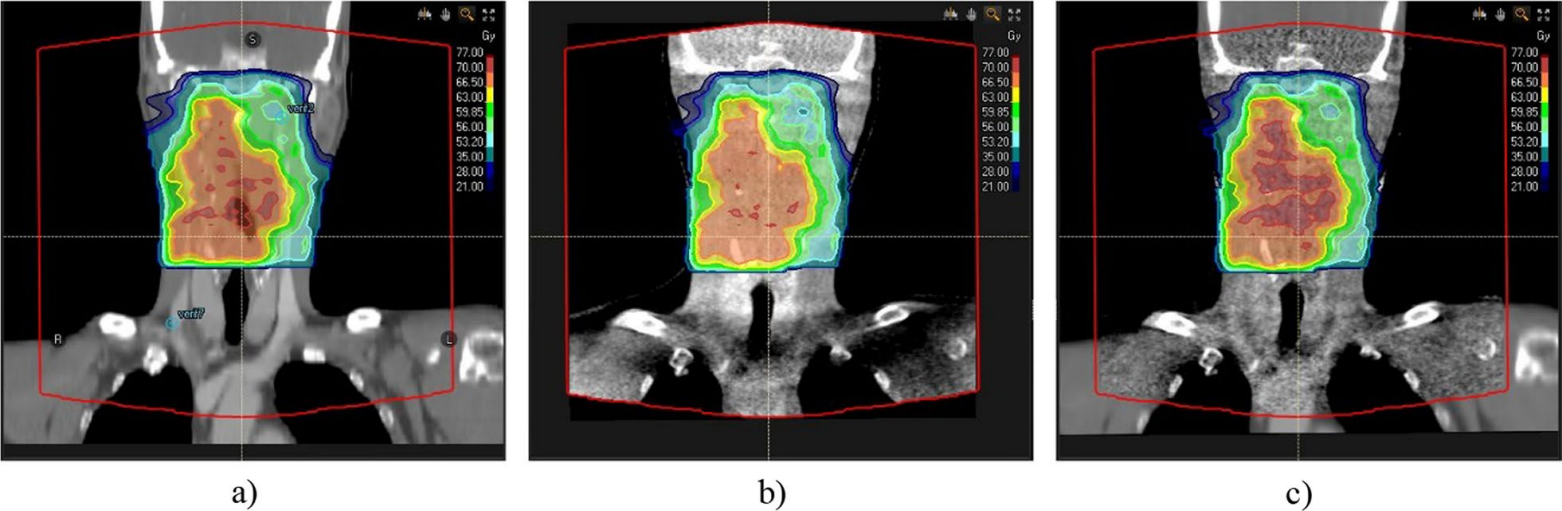
Screenshots of a HN case. The pCT is shown in a), the CBCT_b_ is shown in b) and the CBCT_c_ with the pCT stitched to the regions outside the FOV is shown in c). The FOV of the CBCT is annotated in red

The workflow or the analysis of the data is shown in Fig. 1. For the HN and GYN patients the pCT was rigidly and in a subsequent step deformably registered to the CBCT to generate a deformed CT (dCT) which has similar anatomical features as the CBCT to reduce the influence of anatomical changes between the pCT and the CBCT. The settings for the deformable image registration are shown in Table 1. The same deformable registration was used for generating the dCT and the CBCT_c_. The alignment of the dCT with the CBCT was assessed after the deformable registration and the resulting deformation vector field was visually inspected for unreasonable and particularly large deformations. The generation of the dCT was omitted for the LNG patients since large parts of the body were outside the FOV of the CBCT and therefore introduced large artefacts while deforming the CT. Thus, all LNG patients were only analyzed using the pCT as ground truth. The CBCT_c_ and the CBCT_b_ were generated for all patients using the respective algorithms. For each patient in the HN group, the original treatment plan was re-calculated using the pCT, dCT, CBCT_c_ and the CBCT_b_ within the region of the body fully covered by the FOV of the CBCT as “External” contour ﻿(see Fig. 2). For each patient in the GYN and LNG group, the original treatment plan was re-calculated using the pCT, dCT, CBCT_c_ and the CBCT_b_ with the FOV of the CBCT as “External” contour. This means that dose was calculated in the same region for each image set and therefore allows a fair comparison of both CBCT conversion methods. No further optimization of the treatment plan was performed.

**Table 1:** Parameters used for the deformable image registration between pCT and CBCT. The default settings implemented in the TPS were used in this study

Parameter	Value
Deformation strategy	Default
Similarity measure	Correlation coefficient
Deformation grid resolution	0.25 cm × 0.25 cm × 0.25 cm
Focus ROI	CBCT FOV contracted by 2 cm

*ROI* Region of interest, *FOV* field of view

To assess the performance of the stitching technique, the dose calculated on the pCT and the CBCT_c_ was recalculated for the HN and GYN cases using the original body contour as external contour. The LNG cases were excluded from this evaluation as the beam entrance and the target volume were not affected by the FOV of the CBCT).

All dose files were exported and analyzed using the MICE toolkit (NONPIMedical AB Sweden, Umeå). Gamma analysis was performed for four different dose threshold levels: 10%, 30%, 50% and 90%, relative to the prescribed dose, performing local gamma analysis with a 1% dose difference and a 1 mm distance to agreement criterion. The resulting gamma pass rate (GPR) defined as the percentage of gamma indices smaller than or equal 1 was recorded.

The differences between the investigated groups were analyzed statistically using the Wilcoxon rank sum test considering a *p* value smaller than 0.05 as statistically significant. Statistical computing was performed using R (V. 4.2.2, R Foundation for Statistical Computing, Vienna, Austria).

## Results

**Fig. 3:**
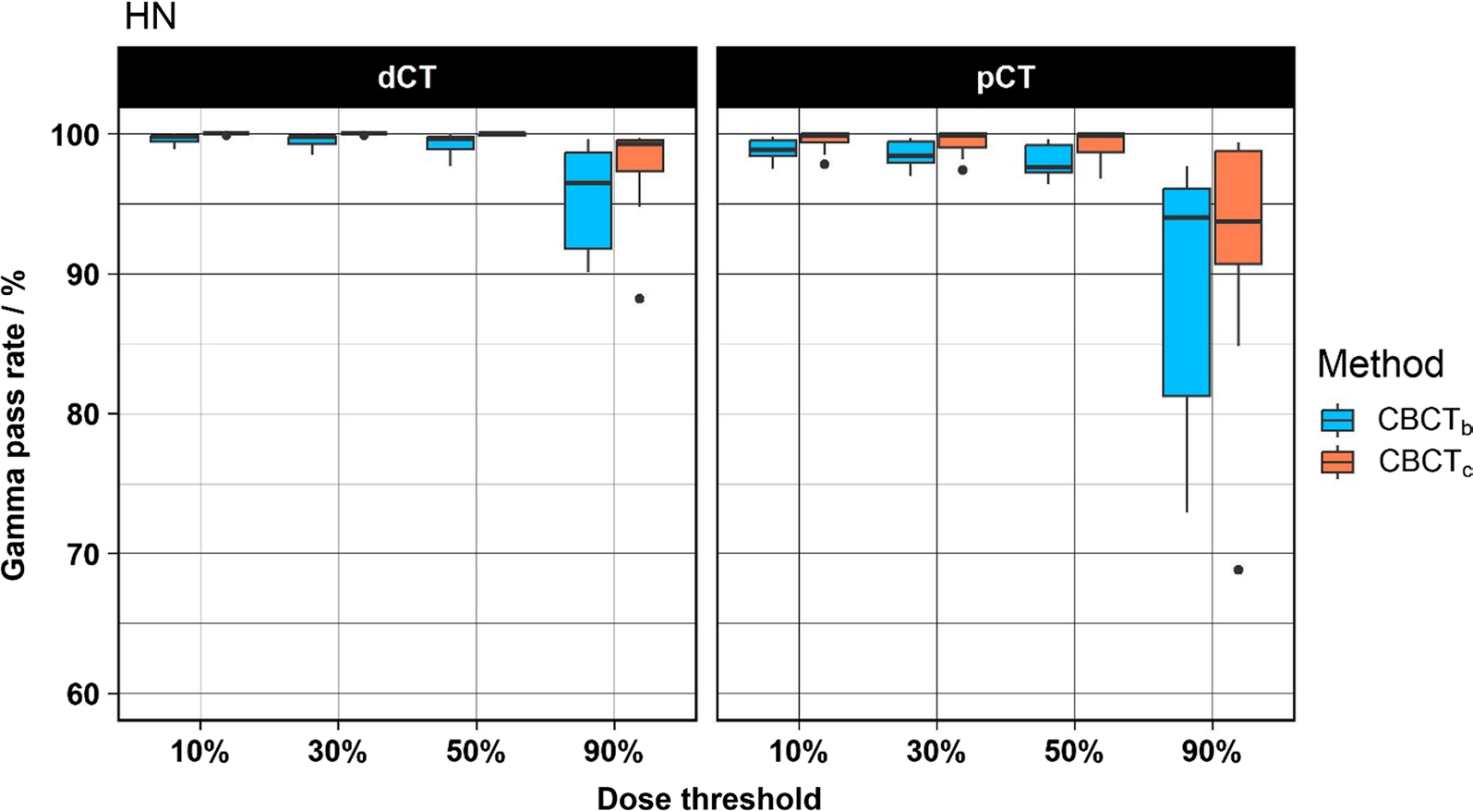
Boxplot of the results of the HN cases. The results using the dCT as reference are shown on the left and the results using the pCT as reference are shown on the right

The results of the HN cases are shown in Fig. 3. The CBCT_c_ groups show systematically higher GPRs compared to the CBCT_b_ group. On average, the GPRs were close to 100% for the CBCT_c_. The differences between the two groups were statistically significant for the dose thresholds 10%, 30% and 50%. No significant difference between the two techniques were found using the 90% dose threshold. Using the dCT as reference at the 90% dose threshold, the median GPRs were 99.3% and 96.5% for CBCT_c_ and CBCT_b_, respectively.

**Fig. 4:**
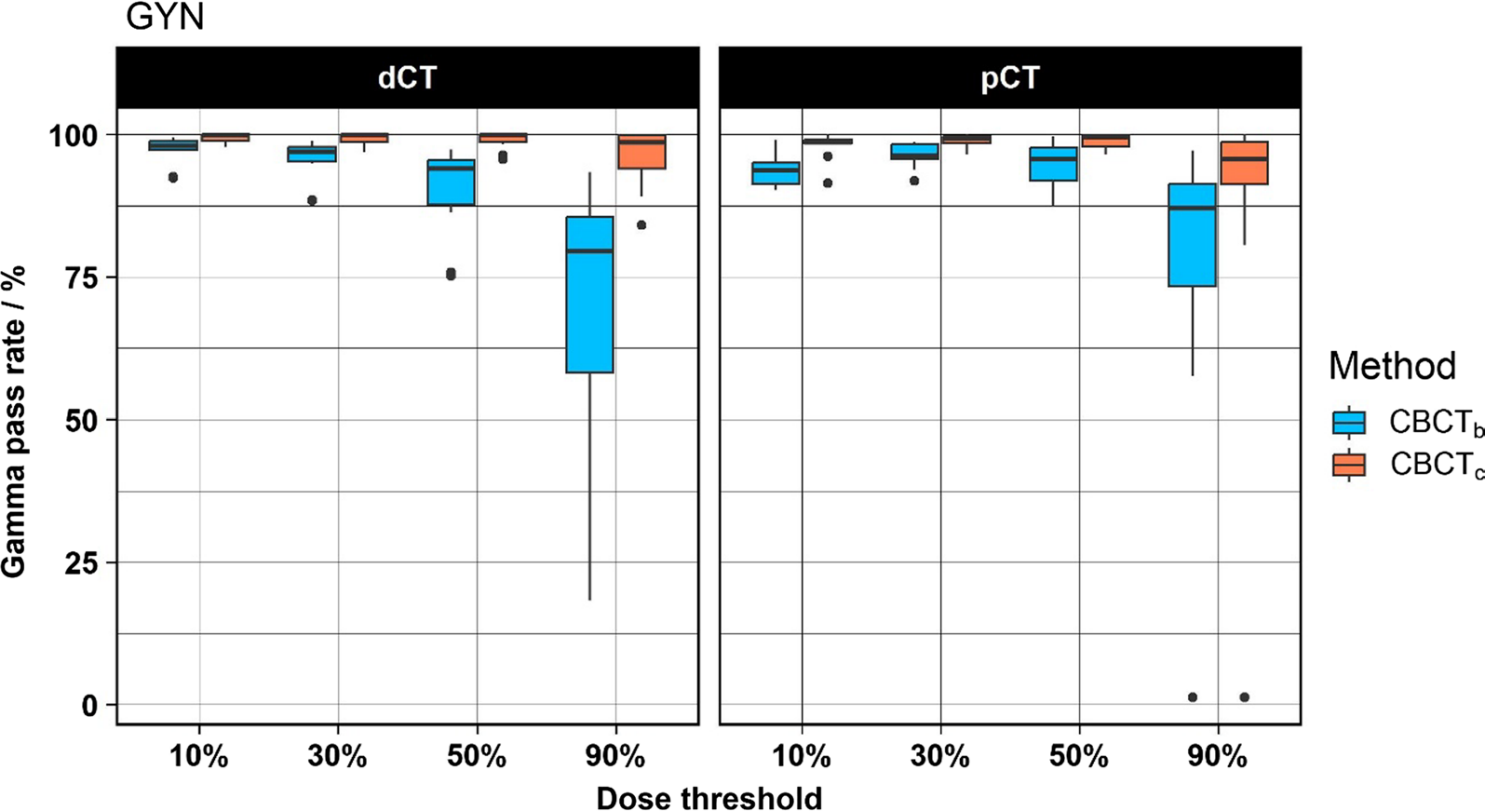
Boxplot of the results of the GYN cases. The results using the dCT as reference are shown on the left and the results using the pCT as reference are shown on the right

Larger differences were found for the GYN cases. Using the dCT as reference, the CBCT_c_ method showed significantly higher GPRs compared to the CBCT_b_ method for all thresholds (Fig. 4). The median GPR was 98.7% and 79.5% at the 90% threshold for the CBCT_c_ and CBCT_b_ group, respectively. Using the pCT as reference, the GPRs were also significantly higher for the CBCT_c_ group for all thresholds except the 90% threshold. One case in this group showed a GPR close to 0% using the pCT as reference for both methods. The iso dose distribution of this case is shown in Additional file 1: Fig. S1. In that case, the patient had a substantial amount of air in the rectum during the pCT which was not present in the CBCT and distorted the dose distribution of the PTV and caused this low GPR.

**Fig. 5:**
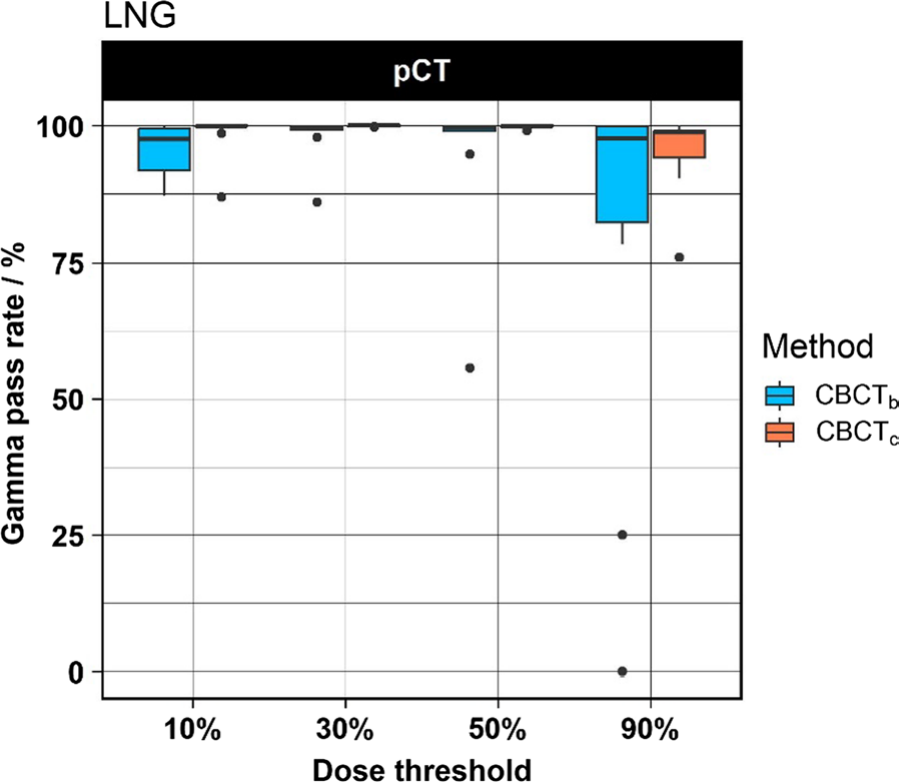
Boxplot of the LNG cases. Note that, the generation of a dCT was not possible for these cases. Therefore, only the results of the pCT are shown

Since it was not possible to generate a reasonable dCT for the LNG patients, only the pCT was used as reference. The results of the LNG cases are shown in Fig. 5. Similar to the previous indications the CBCT_c_ group showed systematically higher GPRs compared to the CBCT_b_ group. The differences were statistically significant for the 10% and 30% threshold. The median GPRs using the 90% threshold were 97.7% and 98.8% for the CBCT_b_ and CBCT_c_ group, respectively.

**Fig. 6:**
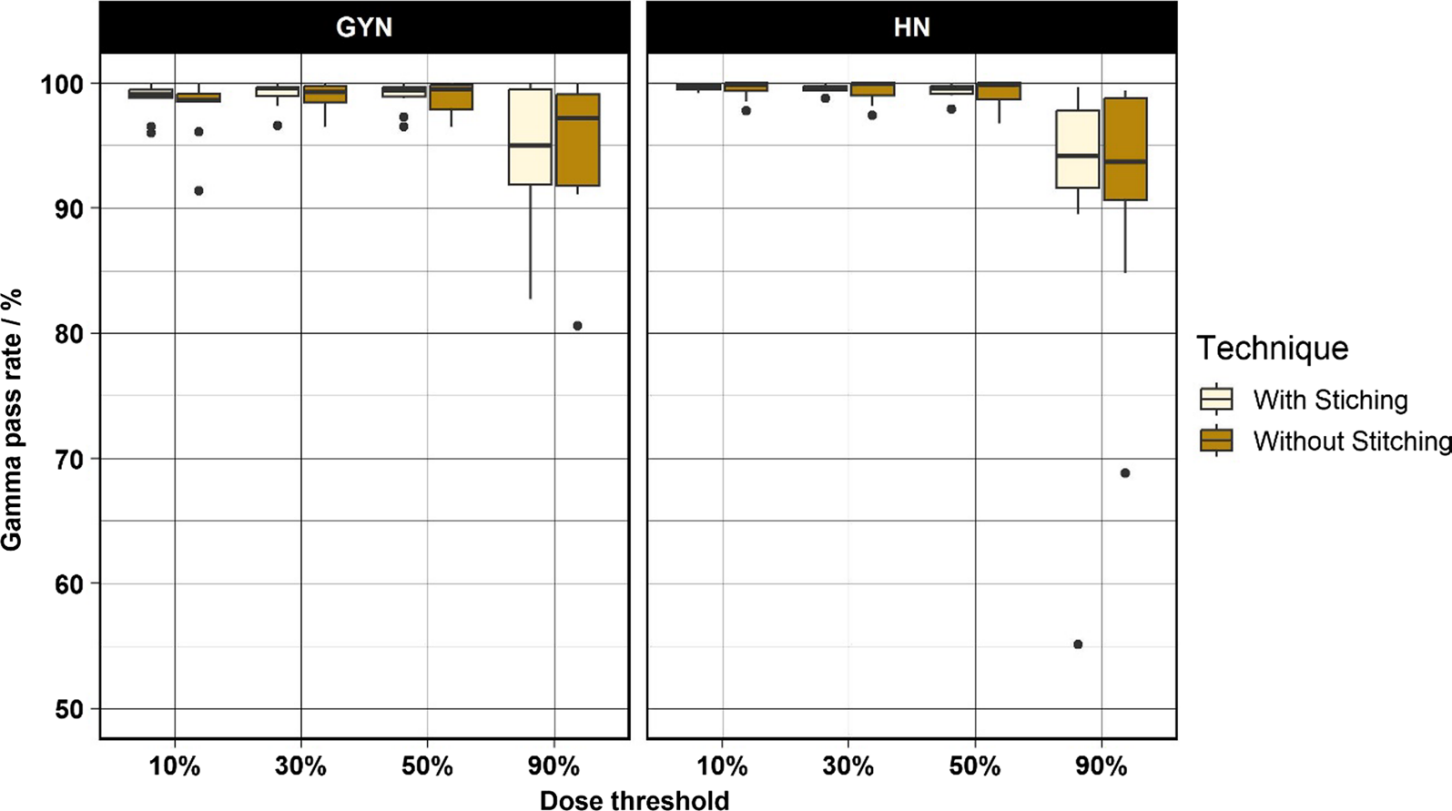
Boxplot of the results of the evaluation of the stitching technique applied using by the CBCT_c_ conversion technique

The results of the evaluation of the stitching technique are shown in Fig. 6. The median GPR of the stitching group was 99.8% and 99.1% at the 10% threshold for HN and GYN group, respectively. The median GPR of the no-stitching group was 99.9% and 98.7% at the 10% threshold for HN and GYN group, respectively. No significant difference between the stitching and no-stitching groups were found for the investigated indication and thresholds.

## Discussion

In this work, the differences between the dose calculated using a novel method was compared to the standard method available in a research version of the TPS RayStation. The results showed superiority of the new CBCT_c_ method compared to the standard CBCT_b_ method, which was statistically significant for all dose threshold except the 90% threshold.

Interpreting the results of 3D gamma analysis is often challenging as regions failing the acceptance criterion are often overcompensated by large regions of passing voxels in other regions. Evaluating the gamma indices for different thresholds allows focusing on specific regions of the dose distribution. For example, using the 90% threshold focuses the evaluation of the gamma analysis on the high dose region surrounding the PTV. This allows to identify regions where the agreement between the evaluated and the reference dose distribution exceeded the acceptance criteria which were not detected using lower thresholds. As shown in Fig. 5, the spread of GPR was larger for the CBCT_b_ method compared to the other indications when using the same threshold. This can be explained by the fact that the 10% isodose line covers large parts of the lung tissue. The bulk-density-override technique assigns a standard density of 0.26 g/cm^3^ to the lung tissue. As the lung tissue is very heterogeneous, the assignment of a fixed number for the density can cause local over- or underestimation of the density and consequently a lower dose calculation accuracy in this region. An example of this behavior is presented in the Additional file 1: Fig. S3. Regions of failing voxels were often found at tissue air interfaces caused by anatomical variations between the pCT and the CBCT. Although the generated dCT largely compensated these differences, a few regions remained where the anatomical changes were not compensated perfectly. This was caused by the regularization of the employed deformable image registration algorithm which prevents the algorithm from producing unrealistic results. Another example where evaluation of different dose thresholds was useful was the outlier case in the GYN group. In an adaptive radiotherapy workflow automated evaluation processes are necessary to highlight whether a plan adaptation is necessary. Using the gamma analysis at different dose thresholds or restricting the gamma analysis to specified organs can serve this purpose.

An additional benefit of the novel CBCT_c_ method is the stitching of the pCT to regions outside the FOV of the CBCT. This allows a simulation of the tissue not covered by the CBCT. The resulting dose distributions in this region need to be interpreted carefully as the regions outside the FOV of the CBCT are also affected by anatomical changes. Nevertheless, the stitching feature is a helpful functionality for treatment simulation.

In general, our results are consistent or even exceed findings in the literature. Note that, in this work an acceptance criterion of 1% / 1 mm was used which is tighter compared to results reported in the literature [13–21]. Thing et al. conducted a similar investigation to this work using the previous version of the TPS and different anatomical regions. In the male pelvis region, they reported GPRs higher than 98% using a 2%/2 mm acceptance criterion, which can be confirmed by our work where similar GPRs were observed with an even tighter acceptance criterion. In our work we focused solely on the gamma analysis using different thresholds as it is independent of contouring. Other groups frequently use the comparison of DVH parameters contoured on the pCT and the CBCT. The contours on the CBCT are usually created either by mapping the contours from the pCT to the CBCT, or by manual contouring. This can create an additional variation of the DVH parameters due to the different anatomy can potentially introduce a bias. Focusing on the gamma analysis avoids this issue.

A limitation of this work is the absence of CT and CBCT data acquired on the same day. This was partially compensated by creating the dCTs having similar anatomical features as the CBCT. Although, the use of CTs acquired on the same day as the CBCT is considered as highest quality ground truth, anatomical changes can also occur between these two image series. In other words, it is rarely possible to acquire a set of identical images in a clinically realistic scenario. Therefore, the approach using the dCT as reference was considered as an adequate substitute. Unfortunately, CBCT data from other vendors were not available. Therefore, the results presented in this work are limited to CBCTs acquired using Elekta XVI. The investigated body sites and presets in this work cover the majority of clinically relevant scenarios and the results are likely transferable to other vendors.

It has been shown, that the image quality of CBCTs can be improved using artificial intelligence [25–28]. A better image quality of the CBCT will further improve the dose calculation accuracy, although the accuracy of the dose calculation of the presented method is already very high. However, enhancing the image quality of CBCTs has the important additional benefit, that the CBCT might be used for contouring in an adaptive radiotherapy workflow as well.

## Conclusion

The novel CBCT_c_ conversion method investigated in this work shows statistically significant improvements compared to the standard CBCT_b_ method implemented in the TPS RayStation considering the 1%/1 mm 10% dose threshold. Regions of failing voxels were often found at tissue air interfaces influenced by anatomical changes. The novel algorithm has clear advantages compared to the standard algorithm and can be introduced into clinical routine.

### Supplementary Information

**Additional file 1: **Supplementary file containing example dose distributions.

## Data Availability

The datasets generated and/or analysed during the current study are not publicly available due to confidentiality and privacy concerns but numerical data are available from the corresponding author on reasonable request.

## Abbreviations

ART: Adaptive radiotherapy
CT: Computer tomography
CBCT: Cone beam computer tomography
CBCT_b_: Cone beam computer tomography with bulk density override
CBCT_c_: Cone beam computer tomography created using the novel correction algorithm
dCT: Deformed CT
DVH: Dose volume histogram
FOV: Field of view
GPR: Gamma pass rate
GYN: Gynecological
HN: Head and neck
iMAR: Iterative metal artifact reduction
LNG: Lung
pCT: Planning CT
PTV: Planning target volume
ROI: Region of interest
TPS: Treatment planning system
