# Supplementary material for: Evaluation of a novel CBCT conversion method implemented in a treatment planning system
Source: Radiat Oncol. 2023 Nov 16;18:191. doi: 10.1186/s13014-023-02378-2 (PMC10655347; doi:10.1186/s13014-023-02378-2)
Supplement: Supplementary file 1 — Additional file 1: Supplementary file containing example dose distributions. [file 13014_2023_2378_MOESM1_ESM.pdf]

Supplementary material GYN case with gas pocket

a) b) c)

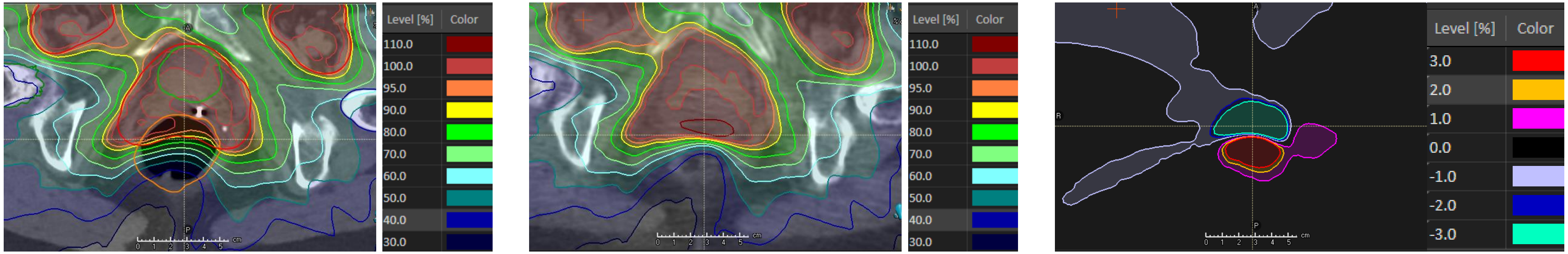

Figure S1 Screenshots of the iso dose distribution of the GYN case with nearly zero GPR. a) shows the pCT with a gas pocket in the rectal canal, b) shows the CBCT with no gas pocket in the same region, c) the dose difference between the two dose distributions in the same region ranging from -3 to +3%.

Supplementary material typical HN case

a) b) c)

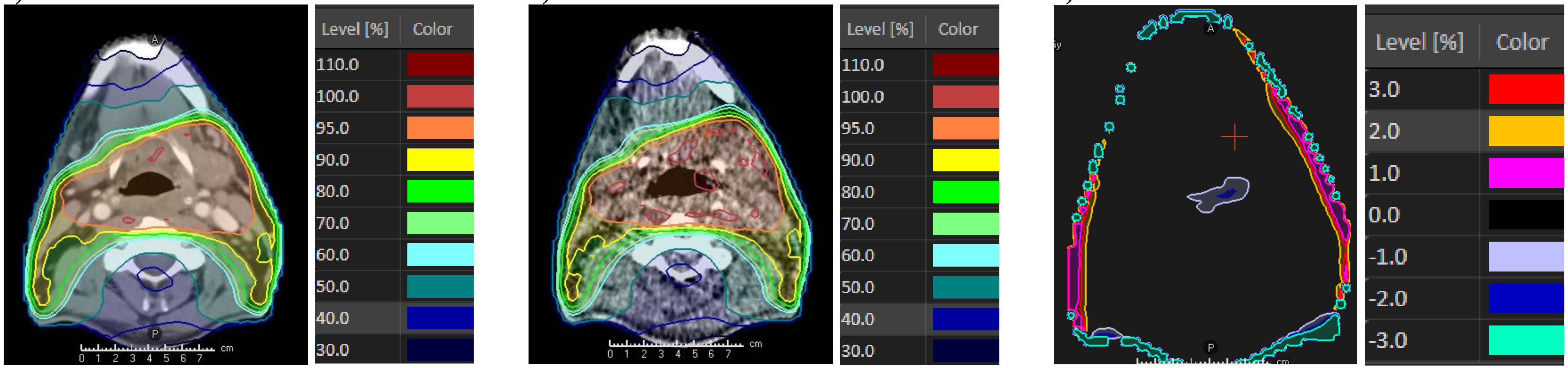

Figure S2 Screenshots of the iso dose distribution of a typical HN case a) shows the pCT, b) shows the CBCT, c) the dose difference between the two dose distributions in the same region ranging from -3 to +3%. The main differences between the two dose distributions were found near tissue-air interfaces as seen at the skin or near the larynx which is wider in the CBCT compared to the pCT.

Supplementary material LNG Case:

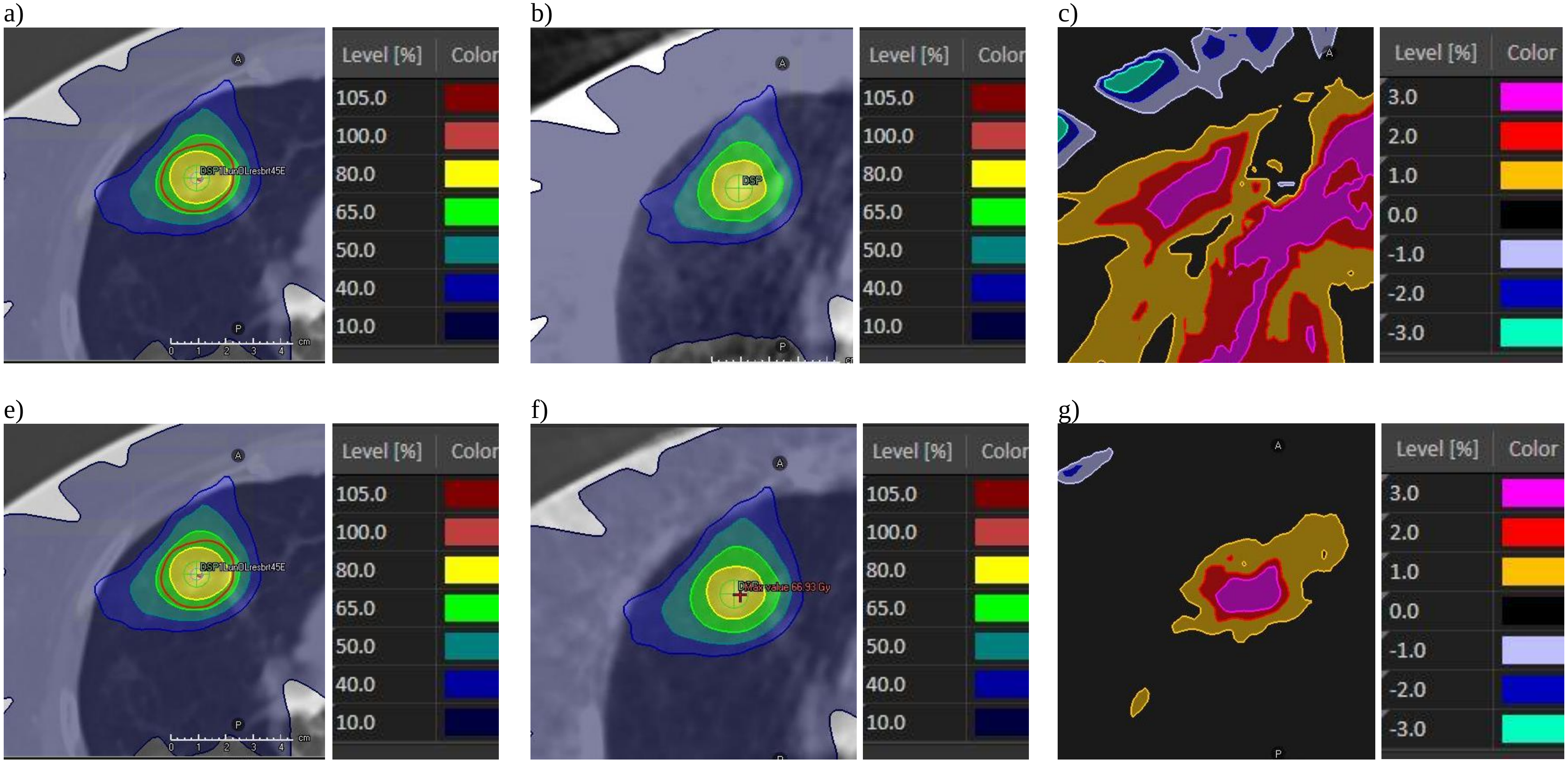

Figure S3 Screenshots of the iso dose distribution of a LNG case a) shows the pCT, b) shows the $CBCT_b$, c) the dose difference between the two dose distributions a and b in the same region ranging from -3 to +3%. e) shows the pCT again, f) shows the $CBCT_c$, g) the dose difference between the two dose distributions e and f in the same region ranging from -3 to +3%. The 10% isodose line is shown in dark blue and covers large parts of the lung. Note, that the accuracy of the dose calculation is higher for the $CBCT_c$ method compared to the $CBCT_b$ method as indicated in c and g.
